# DNA Fragments Assembly Based on Nicking Enzyme System

**DOI:** 10.1371/journal.pone.0057943

**Published:** 2013-03-06

**Authors:** Rui-Yan Wang, Zhen-Yu Shi, Ying-Ying Guo, Jin-Chun Chen, Guo-Qiang Chen

**Affiliations:** 1 MOE Key Lab of Bioinformatics and Systems Biology, Department of Biological Science and Biotechnology, School of Life Sciences, Tsinghua-Peking Center for Life Sciences, Tsinghua University, Beijing, China; 2 Center for Nano and Micro Mechanics, Tsinghua University, Beijing, China; 3 Synthenome.com, Dingley Village, Victoria, Australia; Louisiana State University and A & M College, United States of America

## Abstract

A couple of DNA ligation-independent cloning (LIC) methods have been reported to meet various requirements in metabolic engineering and synthetic biology. The principle of LIC is the assembly of multiple overlapping DNA fragments by single-stranded (ss) DNA overlaps annealing. Here we present a method to generate single-stranded DNA overlaps based on Nicking Endonucleases (NEases) for LIC, the method was termed NE-LIC. Factors related to cloning efficiency were optimized in this study. This NE-LIC allows generating 3′-end or 5′-end ss DNA overlaps of various lengths for fragments assembly. We demonstrated that the 10 bp/15 bp overlaps had the highest DNA fragments assembling efficiency, while 5 bp/10 bp overlaps showed the highest efficiency when T4 DNA ligase was added. Its advantage over Sequence and Ligation Independent Cloning (SLIC) and Uracil-Specific Excision Reagent (USER) was obvious. The mechanism can be applied to many other LIC strategies. Finally, the NEases based LIC (NE-LIC) was successfully applied to assemble a pathway of six gene fragments responsible for synthesizing microbial poly-3-hydroxybutyrate (PHB).

## Introduction

The assembly of pathways, controllable systems and whole genomic level manipulation are important in synthetic biology and for certain applications including microbial productions of antibiotics, biofuels, biomaterials and the creation of minimal free living cells [1], [2], [3], [4]. As a result, the need for efficient manipulation of many genes and large DNA fragments has become an important issue [5], [6], [7]. Although some traditional cloning methods are widely used and or are modified to fit this need [8], the restriction endonucleases digestion and DNA ligase ligation based methods do not permit seamlessly assembling multiple DNA fragments at the same time [9].

A number of new cloning technologies have been developed [10]. Among them, methods of ligation-independent cloning (LIC) such as LIC based on exonuclease [9], [11], sequence and ligation independent cloning (SLIC) [12], improved SLIC (i.e. the one-step thermo-cycled assembly method [13]), and uracil excision-based cloning [14]–[18], have become popular. All these LIC methods are based on the annealing of complementary single-stranded (ss) DNA [9]. DNA exonucleases such as T4 DNA polymerase or lambda exonuclease, have been used to produce single-stranded overlaps as described in most of the above methods except the uracil excision-based cloning one [19].

However, the chew-back with DNA exonuclease led to the formation of uncontrollable lengths of ss DNA overlaps [11], [12]. Generally, the length of the generated ss overlap could be roughly estimated from the duration of DNA exonuclease treatment [19]. It was reported that a 5 min chew-back using DNA exonuclease was sufficient to generate ss DNA overhangs less than 80 bp, while the detailed length distribution was still unknown [13]. Uracil excision–based cloning is a method producing a controllable length of the single-stranded overlap [14], [20]. It adopts uracil-DNA glycosylase (UDG) to treat the uracil bases incorporated into the DNA strand by using uracil containing PCR primers.

These new methods have enabled the seamless cloning of DNA, further allowed the synthesis of large genomic DNA fragments and eventually the bacterial genome [13]. However, in cases of manipulating large DNA fragment systems, cost and efficiency are sometimes more important than the seamlessness. The uncontrollable length distribution of overlapping DNA sequence in SLIC can possibly decrease the efficiency of multiple DNA fragments assembly. There are also some limitations for the USER-LIC method (Uracil-Specific Excision Reagent-LIC): first, it can only be applied to PCR products; second, only 3′-end single-stranded overhangs can be produced due to the single deoxyuridine (dU) placed in the 5′-end of the fragment; third, the synthesis of the dU containing DNA fragment is at high-cost, only specific polymerases which incorporate a deoxyadenine opposite to a dU can be used for DNA amplification [14].

Nicking endonucleases (NEases) have been known for a long time [21], [22]. Similar to restriction endonucleases, they recognize short specific DNA sequence and digest DNA at a defined sequence position related to the recognition sequences [21]. However, many nicking endonucleases were suggested to be naturally mutated restriction endonucleases without the ability to dimerize [23], [24]. Thus, NEases cleave only one predetermined DNA strand of a double-stranded (ds) DNA [21]. NEases were reported to digest target DNA sequentially for engineering single-stranded DNA suitable for fluorescent labeling through end-filling [25], [26], for internal modification of single-molecules [25], construction of novel ligation-independent cloning methods [27], annealing of complementary DNA sequences [28] and for generating long overhangs [29].

In order to develop a low-cost strategy to generate controllable ss DNA overhangs from all types of DNA substrates, a NEases based LIC (NE-LIC) method that can generate controllable overhangs was developed from this study.

## Results

### NEases based LIC (NE-LIC) Coupled with *in vivo* Circularization

**Figure 1:**
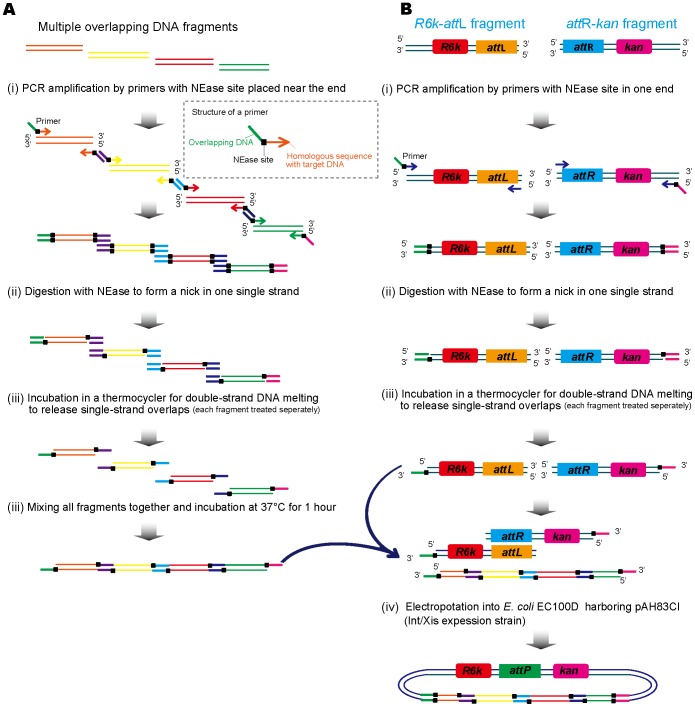
Assembly of multiple DNA fragments based on NE-LIC coupled with *in vivo* circularization. The single-stranded overlaps generated by NEases digestion were annealed *in vitro* and circularized *in vivo* based on *attL/attR* recombination. (A) The procedure of multiple overlapping DNA fragments assembled into a linear form based on nicking enzyme system *in vitro*. (B) The linear DNA circularization procedure *in vivo*.

Single-stranded overlaps annealing generated via nicking enzymes digestion was performed as described in Figure 1A. First, target DNA fragments were amplified with a pair of specific primers. The primers consist of an overlapping sequence, a NEase site and a homologous sequence of the target DNA fragments. Because the long non-homologous sequence was placed in the primer, two cycles of PCR were performed as described in Materials and Methods. Following PCR amplification and DNA purification, all fragments were digested by nicking endonucleases to produce a nick at one single strand of the double-stranded DNA fragments, followed by incubating the digested fragments in a thermo-cycler for formation of single-stranded DNA overlaps via denaturing the double strands DNA at 90°C for 5 min. The concentrations of all the fragments were tested by Nano Drop Spectrophotometer ND-2000 after the incubation. Equal molar DNA fragments containing single-stranded overlaps were mixed together and incubated at 37°C for one hour to anneal all single-stranded overlaps together. During the *in vitro* annealing process, different buffers such as Fast-*pfu* polymerase buffer, T4 DNA polymerase and T4 DNA ligase buffers were screened. The T4 DNA ligase buffer presented the highest efficiency for all ss DNA annealing during the incubation (data not shown).

After the assembly of all DNA fragments *in vitro*, the *in vivo* circularization was performed (Fig. 1B). Two separate fragments were designed as the essential vector skeleton containing an *attL* site with a R6Kγorigin (R6Kγ*ori*) of replication and an *attR* site with a *kanamycin* gene (Fig. 1B). Each of these two fragments was amplified with one end containing a NEase site and an overlapping DNA (Fig. 1B), which was used to anneal with the other DNA fragments. The *attL* and *attR* sites of the essential vector skeleton can achieve site-specific recombination with the expression of integrase (Int) and excisionase (Xis). Therefore, the linear system (Fig. 1A) of the assembled fragments was circularized through *attL*/*attR* site-specific recombination. The circulation process was used in *E. coli* EC100D *pir*-116 competent cells harboring plasmid pAH83CI for Int/Xis expression (Fig. 1B and Table 1). All the primers used for polymerase chain reaction (PCR) are listed in Table 2.

**Table 1:** Bacterial strains and plasmids used in this study.

Stains/plasmids	Description	Reference
*E. coli* EC100D *pir-*116	F^-^ *mcrAΔ(mrr-hsd*RMS*-mcr*BC*)* Ф80d*lac*ZΔM15 Δ*lac*X74*rec*A1 *end*A1 *ara*D139 Δ*(ara, leu)*7697*gal*U *gal*K λ^-^ *rps*L *nup*G *pir*-116 (DHFR)	[31]
pUC19	Cloning vector, Amp^R^	TaKaRa Bio Inc
pKD3	Template plasmid with Amp^R^ and Cat^R^ genes and FLP recognition target	[32]
pBHR68	*phaCAB* expression plasmid, Amp^R^	[33]
pUKG	R6kγ*ori*, *attL/attR* harboring, Kan^R^	This study
pAH83CI	Helper plasmid expressing phage HK022Int/Xis,Amp^R^	[34]
pUKG68	R6kγori, *Re-phaCAB* harboring, Kan^R^	This study

**Table 2:** PCR primers used in this study.

Primers	Sequence
Nb.BbvCI-3'	
R6KFhomo5	5′-GCT***GC*** ***TGAGG***GCAAGATCCGCAGTTCAACCTG
KANRhomo5	5′-GCA***GC*** ***TGAGG***ATTAGAAGAACTCGTCAAGAAGGCGATA
R6KFhomo10	5′-GCAGTCCG***GC*** ***TGAGG***GCAAGATCCGCAGTTCAACCTG
KANRhomo10	5′-GCCGGACT***GC*** ***TGAGG***ATTAGAAGAACTCGTCAAGAAGGCGATA
R6KFhomo15	5′-GCAGTCCGGGACG***GC*** ***TGAGG***GCAAGATCCGCAGTTCAACCTG
KANRhomo15	5′-GCCGTCCCGGACT***GC*** ***TGAGG***ATTAGAAGAACTCGTCAAGAAGGCGATA
R6KFhomo20	5′-GCAGTCCGGGACGTGGAT***GC*** ***TGAGG***GCAAGATCCGCAGTTCAACCTG
KANRhomo20	5′-GCATCCACGTCCCGGACT***GC*** ***TGAGG***ATTAGAAGAACTCGTCAAGAAGGCGATA
R6KFhomo25	5′-GCAGTCCGGGACGTGGATAACCC***GC*** ***TGAGG***GCAAGATCCGCAGTTCAACCTG
KANRhomo25	5′-GCGGGTTATCCACGTCCCGGACT***GC*** ***TGAGG***ATTAGAAGAACTCGTCAAGAAGGCGATA
Nt.BspQI-5'	
R6KFhomo5	5′-AGTCC ***TGAAGAGC***GCAAGATCCGCAGTTCAACCTG
KANRhomo5	5′-GGACT ***TGAAGAGC***ATTAGAAGAACTCGTCAAGAAGGCGATA
R6KFhomo10	5′-GCAGTCCGGC ***TGAAGAGC***GCAAGATCCGCAGTTCAACCTG
KANRhomo10	5′-GCCGGACTGC ***TGAAGAGC***ATTAGAAGAACTCGTCAAGAAGGCGATA
R6KFhomo15	5′-GCAGTCCGGGACGGC ***TGAAGAGC***GCAAGATCCGCAGTTCAACCTG
KANRhomo15	5′-GCCGTCCCGGACTGC ***TGAAGAGC***ATTAGAAGAACTCGTCAAGAAGGCGATA
R6KFhomo20	5′-GCAGTCCGGGACGTGGATGC ***TGAAGAGC***GCAAGATCCGCAGTTCAACCTG
KANRhomo20	5′-GCATCCACGTCCCGGACTGC ***TGAAGAGC***ATTAGAAGAACTCGTCAAGAAGGCGATA
R6KFhomo25	5′-GCAGTCCGGGACGTGGATAACCCGC ***TGAAGAGC***GCAAGATCCGCAGTTCAACCTG
KANRhomo25	5′-GCGGGTTATCCACGTCCCGGACTGC ***TGAAGAGC***ATTAGAAGAACTCGTCAAGAAGGCGATA
3 fragments Nb.BbvCI-3'	
R6KFhomo15	5′-GCAGTCCGGGACG***GC*** ***TGAGG***GCAAGATCCGCAGTTCAACCTG
KANRhomo15	5′-GCGCATGTAGTAC***GC*** ***TGAGG***ATTAGAAGAACTCGTCAAGAAGGCGATA
catR6K15nb	5′-GCCGTCCCGGACT***GC*** ***TGAGG***ATCCTCCTTAGTTCCTATTCCG
catKAN15nb	5′-GCGTACTACATGC***GC*** ***TGAGG***ATTACACGTCTTGAGCGATTGT
3 fragmentsNt.BspQI-5'	
R6KFhomo15	5′-GCAGTCCGGGACGGC ***TGAAGAGC***GCAAGATCCGCAGTTCAACCTG
KANRhomo15	5′-GCGCATGTAGTACGC ***TGAAGAGC***ATTAGAAGAACTCGTCAAGAAGGCGATA
catR6K15nt	5′-GCCGTCCCGGACTGC ***TGAAGAGC***ATCCTCCTTAGTTCCTATTCCG
catKAN15nt	5′-GCGTACTACATGCGC ***TGAAGAGC***ATTACACGTCTTGAGCGATTGT
6 fragmentsNb.BbvCI-3'	
R6KFhomo15	5′-GCAGTCCGGGACG***GC*** ***TGAGG***GCAAGATCCGCAGTTCAACCTG
KANRhomo15	5′-GCTAAGGTCGTGT***GC*** ***TGAGG***ATTAGAAGAACTCGTCAAGAAGGCGATA
Bref	5′-GCCGTCCCGGACT***GC*** ***TGAGG***CCGGTCGCTTCTACTCCTAT
Brer	5′-GCCCGGTCGCCAT***GC*** ***TGAGG***GATTTGATTGTCTCTCTGCCGTCAC
BphaCf	5′-GCATGGCGACCGG***GC*** ***TGAGG***ATGGCGACCGGCAAAGGCGCGGCAG
BphaCr	5′-GCCACGCTCATGC***GC*** ***TGAGG***TGCCTTGGCTTTGACGTATCGCCCA
BphaBf	5′-GCGCATGAGCGTG***GC*** ***TGAGG***TGACGCTTGCATGAGTGCCGGCGTG
BphaBr	5′-GCGTCACCGTGATA***GC*** ***TGAGG***TTTGCGCTCGACTGCCAGCGCCACG
BphaAf	5′-GCTATCACGGTGAC***GC*** ***TGAGG***TAAGGAAGGGGTTTTCCGGGGCCGC
BphaAr	5′-GCACACGACCTTA***GC*** ***TGAGG***GCCCATATGCAGGCCGCCGTTG
6 fragmentsNt.BspQI-5'	
TR6KFhomo15	5′-GCAGTCCGGGACGGC ***TGAAGAGC***GCAAGATCCGCAGTTCAACCTG
TKANRhomo15	5′-GCGCAtGTAGTACGC ***TGAAGAGC***ATTAGAAGAACTCGTCAAGAAGGCGATA
Tref	5′-GCCGTCCCGGACTGC ***TGAAGAGC***CCGGTCGCTTCTACTCCTAT
Trer	5′-GCCCGGTCGCCATGC ***TGAAGAGC***GATTTGATTGTCTCTCTGCCGTCAC
TphaCf	5′-GCATGGCGACCGGGC ***TGAAGAGC***ATGGCGACCGGCAAAGGCGCGGCAG
TphaCr	5′-GCCACGCTCATGCGC ***TGAAGAGC***TGCCTTGGCTTTGACGTATCGCCCA
TphaBf	5′-GCGCATGAGCGTGGC ***TGAAGAGC***TGACGCTTGCATGAGTGCCGGCGTG
TphaBr	5′-GCGTCACCGTGATAGC ***TGAAGAGC***TTTGCGCTCGACTGCCAGCGCCACG
TphaAf	5′-GCTATCACGGTGACGC ***TGAAGAGC***TAAGGAAGGGGTTTTCCGGGGCCGC
TphaAr	5′-GCGTACTACATGCGC ***TGAAGAGC***GCCCATATGCAGGCCGCCGTTG
Nb.BbvCI with 2 bp non-complementary	
R6KFhomo5	5′-TCCGG ***GCTGAGG***GCAAGATCCGCAGTTCAACCTG
KANRhomo5	5′-CCGGA ***GCTGAGG***ATTAGAAGAACTCGTCAAGAAGGCGATA
R6KFhomo10	5′-TCCGGAACCC ***GCTGAGG***GCAAGATCCGCAGTTCAACCTG
KANRhomo10	5′-GGGTTCCGGA ***GCTGAGG***ATTAGAAGAACTCGTCAAGAAGGCGATA
R6KFhomo15	5′-TCCGGAACCCGGACG ***GCTGAGG***GCAAGATCCGCAGTTCAACCTG
KANRhomo15	5′-CGTCCGGGTTCCGGA ***GCTGAGG***ATTAGAAGAACTCGTCAAGAAGGCGATA
R6KFhomo20	5′-TCCGGAACCCGGACGTGGAT ***GCTGAGG***GCAAGATCCGCAGTTCAACCTG
KANRhomo20	5′-ATCCACGTCCGGGTTCCGGA ***GCTGAGG***ATTAGAAGAACTCGTCAAGAAGGCGATA
R6KFhomo25	5′-AAAGTTCCGGAACCCGGACGTGGAT ***GCTGAGG***GCAAGATCCGCAGTTCAACCTG
KANRhomo25	5′-ATCCACGTCCGGGTTCCGGAACTTT ***GCTGAGG***ATTAGAAGAACTCGTCAAGAAGGCG
*attL/attR* reaction primer	
attLR	5′-CCACATCTTTTCGTTATCGGCAC
attRF	5′-CAGTATGAATCTTTCAGGCTGGGA
Colony PCR primer	
r6kgamma	5′-GCCTCTCAAAGCAATTTTCAGT
tesR	5′-TGTCCAGATAGCCCAGTAGC
r6ktest	5′-ACGTTAGCCATGAGAGCTTAGTAC
k2	5′-CGGTGCCCTGAATGAACTGC
phaBRtest	5′-ACCACACGAAAGCCATCCTT
reFtest	5′-TAGCATCTCCCCATGCAAAG
Primers for cloning fragments used in optimizing denaturing temperature	
BtsIf	5′-CGAGTGGGTTACATCGAACT
BtsIr	5′-TGCACGAACCCCCCGTTCAG

All oligonucleotides were synthesized by Invitrogen (Life technologies, USA). Restriction endonuclease digestion sites are bold italic. Homology sequences are underlined.

### Competing Primers Do Not Increase Denaturation Efficiency

In order to increase denaturation efficiency, competing primers which are identical to the ss DNA overhang were added to a denaturation mixture to function as the competitor for the removal of the complementary strands. However, there was no positive effect observed. In order to remove the competing primers and complementary strands, gel electrophoresis was also employed without positive effect observed either.

### Optimization of the Overhang Lengths

**Figure 2:**
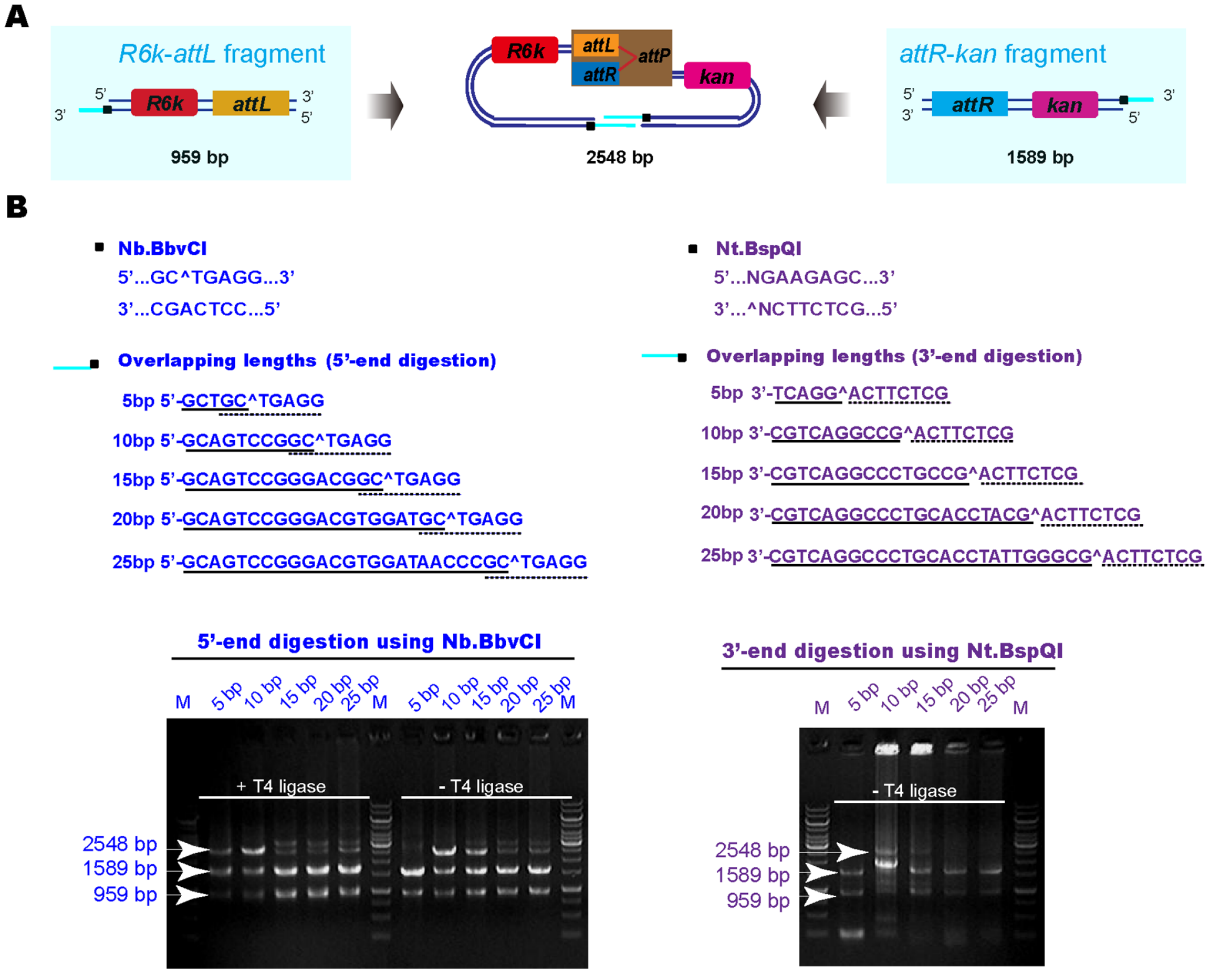
Effects of the overlapping lengths and T4 DNA ligase on fragment assembly using NE-LIC coupled with *in vivo* circularization. (A) Flowsheet of the *R6K-attL* and *attR-kan* fragment assembly. (B) Comparison of the assembly efficiency of different overlapping lengths using both 3′-end single-stranded annealing (5′-end digestion using Nb.BbvCI) with/without T4 DNA ligase (left) and 5′-end single-stranded annealing (3′-end digestion using Nt.BspQI) without T4 DNA ligase (right). The 3′- and 5′-end overlaps of 5 bp, 10 bp, 15 bp, 20 bp and 25 bp generated by Nb.BbvCI/Nt.BspQI were listed. Overlapping base pairs were underlined. NEase sites were underlined with dotted lines. DNA gel electrophoresis verified the assembly results.

With the USER™ cloning method, the dU is excised from the PCR products only at 5′-end of the fragments as dU is designed in each PCR primer [14]. After the USER enzyme digestion, PCR products are flanked by 3′-end ss DNA extensions. Nicking enzymes digestion was conducted either at 3′-end or 5′-end to produce either 5′-end ss DNA or 3′-end ss DNA overlaps (Fig. 2). The Nt.BbvCI cassette (see Materials and Methods) was designed for 5′-end digestion to form 3′-end ss DNA overlaps, while the Nt.BspQI cassette was designed for 3′-end digestion to generate 5′-end ss DNA overlaps (Fig. 2B). The annealing results showed that 3′-end ss DNA overlaps annealed better with other fragments than the 5′-end ss DNA overlaps did, which was attributed to dephosphorylation of the first base at the 5′-end of the primers.

In order to determine the optimal overhang lengths, 5 bp, 10 bp, 15 bp, 20 bp and 25 bp ss overhangs were studied for both 3′-end and 5′-end ss DNA overlaps annealing, respectively. Results of both electrophoresis and chemical transformation showed that the overhangs of 10 bp/15 bp were able to produce the highest cloning efficiency (Fig. 2B and Table 3).

**Table 3:** Transformation results of different lengths of ss overhangs.

	5 bp	10 bp	15 bp	20 bp	25 bp
5′-end digestion with Nb.BbvCI +T4 ligase	23000	10600	5600	1000	1650
5′-end digestion with Nb.BbvCI −T4 ligase	3600	56000	40000	10000	8000
3′-end digestion with Nt.BspQI −T4 ligase	470	2670	5000	900	150

Cloning efficiencies were given as colony forming units per micromole of each fragment. The homology regions were ranged from 5 bp to 25 bp.

### T4 DNA Ligase Enhances Efficiency

T4 DNA ligase was added in order to investigate how it affected the annealing efficiency. When added to an annealing mixture, ligation can be achieved at the annealed single-stranded DNA ends. Since our fragments were prepared by PCR amplification, the 5′-end of the PCR product lacked the phosphate and only 5′ recessed substrate has the phosphate group for ligation. Therefore, when comparing the effect of T4 DNA ligase on 5 bp, 10 bp, 15 bp, 20 bp or 25 bp ss DNA overhangs for 3′-end annealing (5′-end digestion), both results of electrophoresis and transformation revealed that the 5 bp and 10 bp were the best in the presence of T4 DNA ligase. However, in the absence of T4 DNA ligase, 10 bp and 15 bp were found to be optimal both for 3′-end and 5′-end annealing (Fig. 2B). This demonstrated that T4 DNA ligase improved the ligation for short length DNA fragments as T4 DNA ligase favors to link short sticky ends digested by Type II restriction enzymes [8]. For longer single-stranded DNA extensions, the homologous annealing was preferred compared with short single-stranded DNA extension, and both 3′-end as well as the 5′-end ss DNA overlaps annealing results demonstrated the length of a homologous tail of 10 bp and 15 bp had the highest efficiency (Fig. 2B).

### Seamless Annealing Enhanced the Assembly Efficiency

**Figure 3:**
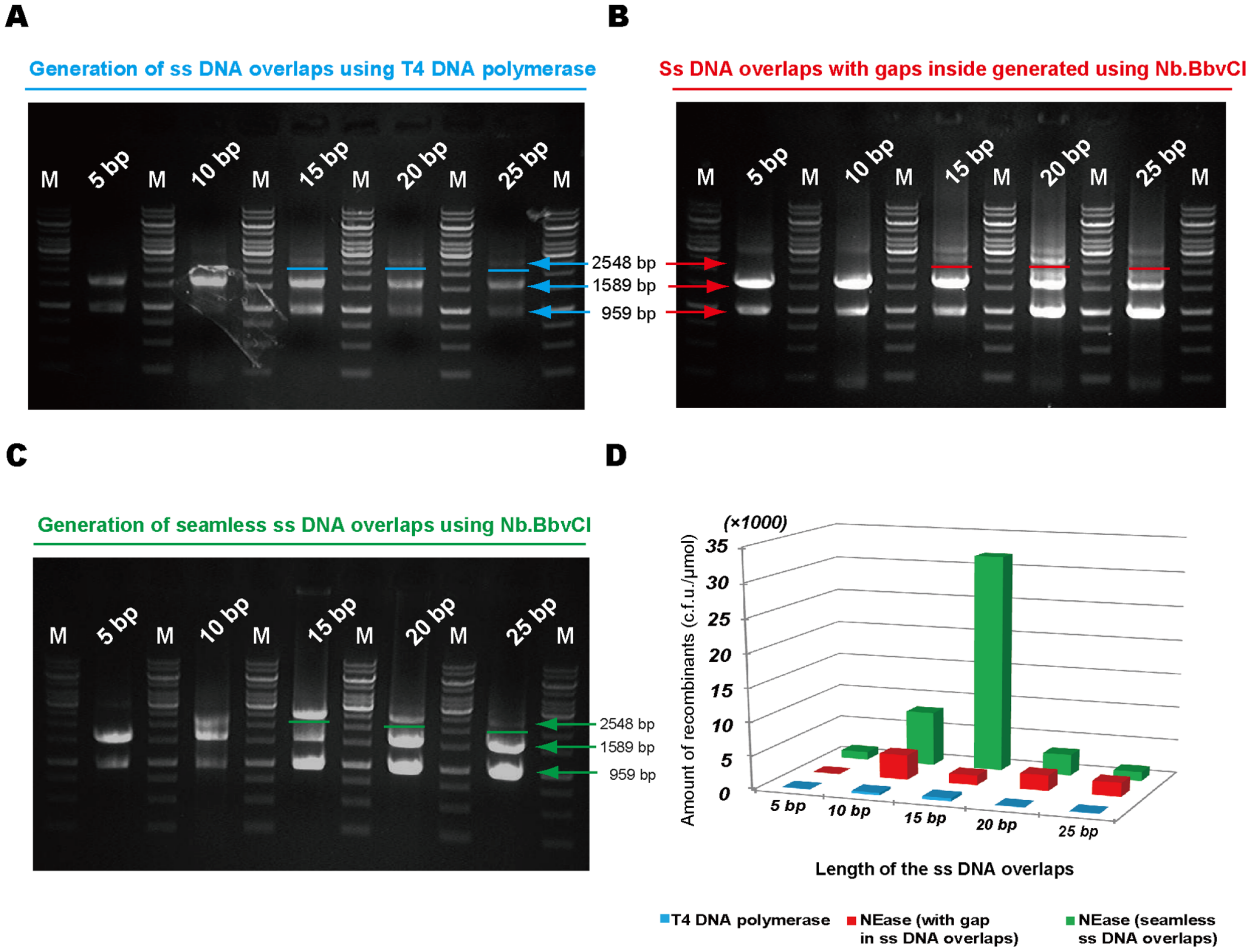
Effects of non-complementary nucleotides in the single-stranded DNA tails on assembly efficiency. Three groups of ss DNA overlaps generation methods: (A) Generation of ss DNA overlapping tails using T4 DNA polymerase led to uncontrollable lengths of the non-complementary nucleotides besides the homologous parts; (B) Nb.BbvCI based generation of ss DNA overlaps with two non-complementary nucleotides designed at the inside end as a small gap; (C) Formation of seamless ss DNA overlaps using Nb.BbvCI. The homologous overlaps were designed with lengths of 5 bp, 10 bp, 15 bp, 20 bp, and 25 bp, respectively. DNA gel electrophoresis of (A), (B) and (C) verified the assembly results. (D) The successful recombinants resulted from of the three (A), (B) and (C) groups, respectively.

Most of the LIC methods rely on the single-stranded overlaps annealing [9]. Since the lengths distribution of ss DNA overlapping tails generated by T4 DNA polymerase (in the absence of dNTP) treatment were unknown [11], [12], and the accurate length of the ss DNA overlaps can be produced using NEases, the comparison of treatments using T4 DNA polymerase and NEases on ss DNA overlaps annealing efficiency was performed (Fig. 3). To study whether gaps generated by an uncontrollable digestion of T4 DNA polymerase (in the absence of dNTP) can result in a decreased efficiency, three groups of assembling studies were conducted (Fig. 3). The first group used T4 DNA polymerase to generate ss DNA overlapping tails; the second one employed nicking endonuclease to form ss DNA overlaps consisting of a two base pairs non-overlap (2 bp gaps); the third one adopted a nicking endonuclease to generate a seamless ss DNA overlaps (Fig. 3). The homologous overlaps from each group were designed with lengths of 5 bp, 10 bp, 15 bp, 20 bp, and 25 bp, respectively. After the T4 DNA polymerase or NEases treatments and further incubation at 37°C, electrophoresis of all mixtures was performed to study the annealing effects (Figs. 3A, 3B and 3C). Results of the chemical transformation showed that the lowest efficiency (Figs. 3A and 3D and Table 4) was from T4 DNA polymerase treatment, which might produce non-complementary ss DNA inside of the ss tail besides the homologous ss overlap, while better results were observed from nicking enzyme treatment with 2 bp gaps (Figs. 3B and 3D and Table 4). On the other hand, NE-LIC without gaps produced the highest efficiency results (Figs. 3C and 3D and Table 4). All phenomena demonstrated that a controllable overhang length enabled higher assembly efficiency.

**Table 4:** Comparison of cloning efficiencies of three methods producing different single-stranded overlaps.

	5 bp	10 bp	15 bp	20 bp	25 bp
T4 DNA polymerase	50	350	450	50	50
NEase (with 2nt gap in ss overlaps)	0	3690	1470	2340	2070
NEase (without gaps in ss overlaps)	1170	8070	32000	3150	1350

Cloning efficiencies were given as colony forming units per micromole of each fragment. The homology regions were ranged from 5 bp to 25 bp.

### Multiple-fragments Assembly Using NE-LIC

**Figure 4:**
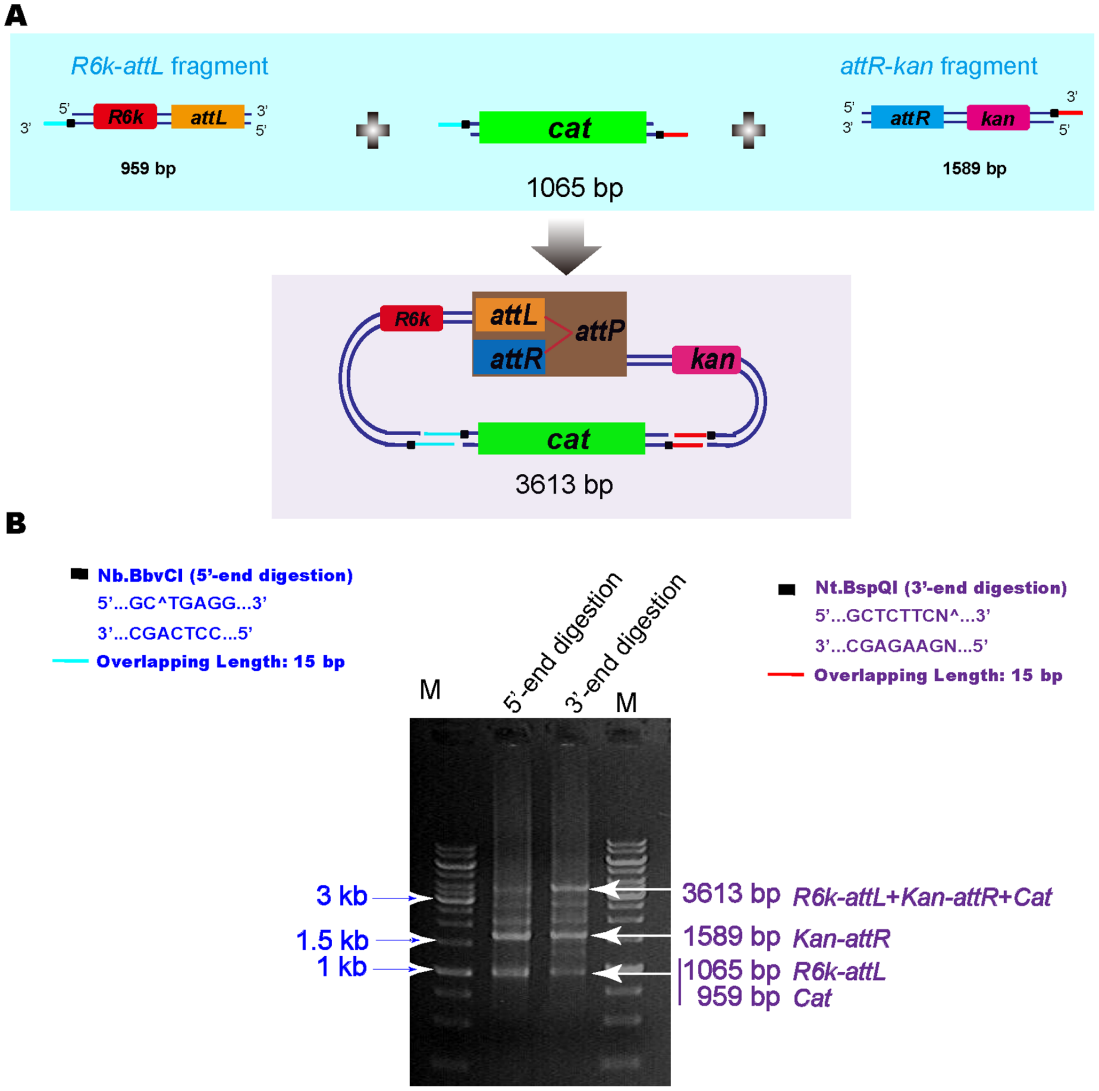
Assembly of three DNA fragments using NE-LIC coupled with *in vivo* circularization. (A) Schematic diagram of the three DNA fragments assembly. (B) Three fragments assembly using both 3′-end (5′-end digestion using Nb.BbvCI) (left) and 5′-end single-stranded (3′-end digestion using Nt.BspQI) (right) annealing. DNA gel electrophoresis verified the assembly results (B-middle).

Fragments of the *attL*-*R6K*, *attR*-*kan*, and *cat* encoding chloromycin, respectively, were assembled using 15 bp single-stranded overlaps (Fig. 4). The gel electrophoresis displayed successful assembly of the three fragments. The reaction mixture was plated on Kan^R^+Cm^R^ Petri disks. 248 colonies that were results of the successful 3′-end ss DNA overlaps annealing, were observed on Kan^R^+Cm^R^ Petri disks. In comparison, for 5′-end ss overlaps annealing, 476 colonies were grown on Kan^R^+Cm^R^ Petri disks. All of the colonies on Kan^R^+Cm^R^ Petri disks contained the *cat* selection marker. The colonies were verified via DNA sequencing, all indicated a correct assembly. In order to investigate the assembly efficiency, the reaction mixture was plated on Kan^R^ Petri disks at the same time. 287 and 532 colonies were found for the 3′ and 5′-end ss overlaps annealing, respectively.

### PHB Synthesis by the Assembled *phaCAB* Operon

**Figure 5:**
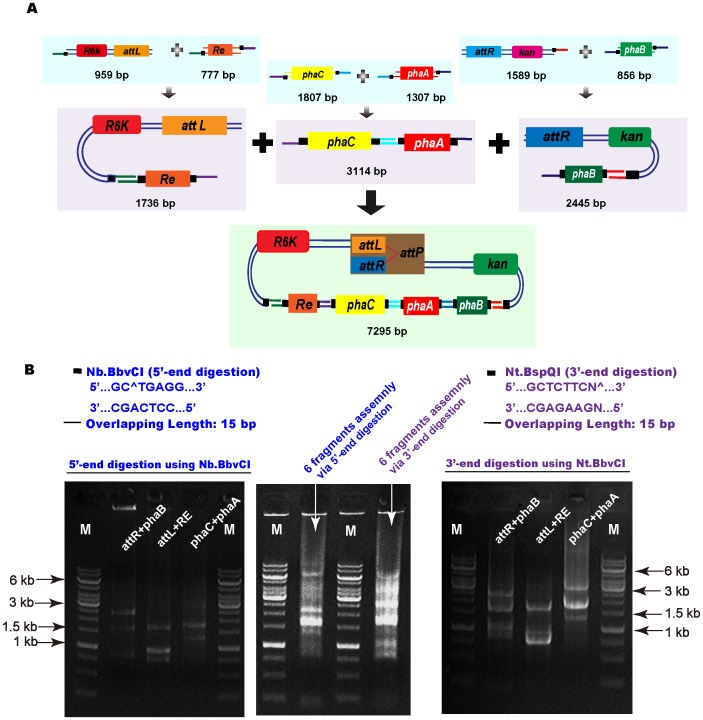
Construction of PHB synthesis pathway via six DNA fragments assembly. (A) Schematic diagram of assembling the six DNA fragments. Each two fragments were assembled first; subsequently the three groups of two-fragments were mixed together. (B) Assembling results using 3′-end and 5′-end single-stranded DNA annealing. Gel electrophoresis of the initial assembly of two DNA fragments using 3′-end ss DNA annealing (5′-end digestion using Nb.BbvCI) was presented on left, and 5′-end ss DNA annealing (3′-end digestion using Nt.BspQI) on right. Middle gel picture corresponded to the six fragments assembly as indicated in the picture.

Poly-3-hydroxybutyrate (PHB) synthesis pathway cloned from *Ralstonia eutropha* was assembled using 15 bp single-stranded overlaps annealing (Fig. 5). Six DNA fragments including *attL*-*R6K* fragment, *attR*-*kan* fragment, *R. eutropha* native promoter (*Re* promoter), genes of beta-ketothiolase (*phbA*), acetoacetyl-CoA reductase (*phbB*) and PHA synthase (*phbC*), were amplified with specific primers containing 15 bp overlaps. The fragments were digested either by the Nt.BbvCI (3′-end ss DNA overlaps) or by the Nt.BspQI (5′-end ss DNA overlaps). After the denaturation process, the incubation led to formation of the ds linear DNA fragment assembled by the above six fragments. They were transformed into *E. coli* EC 100D *pir*-116 harboring pAH83CI.

When assembled all six fragments together in one reaction at 37°C, no colony containing the correct assembled fragments was found. Thus, a different approach was adopted by first incubating two fragments from the six fragments together, such as fragments of the *attL*-*R6K* and *Re* promoter, fragments of genes of of PHB synthase (*phbC*) and beta-ketothiolase (*phbA*), and fragments of *attR*-*kan* and gene of acetoacetyl-CoA reductase (*phbB*) (Fig. 5A). Consequently, the three fragment groups each containing two fragments were mixed together and incubated at 37°C for another half an hour. The expected assembly was transformed into the *E. coli*. Several colonies were observed on the Petri disks after 24 h. PCR verification based on primers phaBRtest/reFtest confirmed all the three randomly picked colonies to have the correct assembly of the six fragments mentioned above (Table 2). As a result, PHB accumulation was detected in the three positive recombinants (Table 5). The three recombinants grew to over 13 g/L cell dry weight (CDW) containing over 40% PHB in their CDW in 48 h of growth, demonstrating the success of the multiple-fragments assembly using NE-LIC.

**Table 5:** PHB production from *E. coli* strains constructed by six fragments NE-LIC.

Recombinant strains	CDW (g/L)	PHB (wt%)	PHB (g/L)
*E. coli* PHB1	13.39±0.38	49.35±2.50	6.61±0.45
*E. coli* PHB2	13.82±0.19	42.74±1.78	5.90±0.17
*E. coli* PHB3	13.56±0.22	43.74±1.16	5.93±0.20

The three randomly picked positive recombinants harboring pUKG68 were cultivated in Terrific Broth (TB) medium containing 20 g/L glucose at 37°C for 48 h as described in “Materials and Methods”. Data shown were the average and standard deviations of three parallel experiments. CDW, cell dry weight.

## Discussion

Although NEases have been used to generate ss DNA overlaps for cloning DNA fragments into plasmids [27], this study is the first one using NEases in ligation-independent cloning (LIC) for constructing a pathway consisting of multiple genes. The assembly of multiple overlapping DNA fragments into a linear form was made by NEases generated single-stranded overlaps annealing *in vitro,* the subsequent circularization of the linear DNA by *attL/attR* recombination *in vivo* led to the successful expression of the assembled genes (Fig. 1).

Both NE-LIC [DNA ligation-independent cloning (LIC) based on Nicking Endonucleases (NEases)] and USER-LIC (LIC based on Uracil-Specific Excision Reagent) adopt nicking strategy to produce ss DNA overhangs [15], [27], it is thus important to study the ss DNA generated processes. Since the nicking enzymes display a nicking activity but no cutting activity of an endonuclease, a strand-specific nick can be generated after the digestion using nicking enzymes, while the phosphodiester backbone still leaves intact with a nick at the digested site [21]. During a denaturation process under a high temperature, the double-stranded DNA was unwound and separated into single-stranded DNA. As a result, one fragment was split into two fragments with single-stranded tails from the nick. In order to test the temperature dependence of splitting the DNA fragment, a fragment from plasmid pUC19 harboring two Nb.BtsI sites with 22 bp interval length was used (See Materials and Methods). Various denaturation temperatures from 70°C to 95°C were used to test the melting effect on generating single-stranded DNA overlaps. The double-stranded DNA fragments were not separated completely at the temperatures below 90°C. Nevertheless, a temperature of 95°C showed a negative effect on the degradation of the double-stranded DNA. The 22 bp DNA gap of two NEase sites could split 90% of the fragment into two ds DNA with ss DNA tails just after the digestion at 37°C, further denaturation at 90°C for 5 min enhanced the split close to 100%. Therefore, a denaturation process at 90°C for 5 min after digestion was recommended as a denaturation optimum. Since the NEase digestion could happen at the NEase sites existed inside the target fragments that could generate disrupted DNA fragments, these NEase sites should be avoided in the design of the primers used for the multiple DNA assembly.

The optimal overlapping length for NEases based LIC (NE-LIC) without T4 DNA ligase was between 10 bp and 15 bp (Fig. 2B and Table 3). This is possible due to the incomplete denaturation of longer ss DNA overlaps and less secondary structures formed from shorter ss DNA overlaps. However, the optimal overlapping length shifted to between 5 bp to 10 bp when T4 DNA ligase was added (Fig. 2B), indicating that the ligation process was more efficient than the annealing one. It is important to emphasize that the annealing of 10 bp/15 bp does not necessarily require T4 DNA ligase (Fig. 2B). Considering the cloning efficiency, the presence of T4 DNA ligase to the NE-LIC system is still recommended.

The NE-LIC demonstrated an improved cloning efficiency compared with other methods including SLIC and the incomplete PCR approach [10], [12]. The unknown length distribution of ss DNA overlaps in SLIC method produced non-complementary gaps in the ss DNA tails, resulting in decreased annealing productivity (Fig. 3). The improved SLIC method using T5 polymerase that fill the DNA gaps helps increase the cloning efficiency [13]. A significant reduced annealing and cloning efficiency were observed when 2 bp gaps were designed in the overlapping DNA in our NE-LIC (Fig. 3 and Table 4). The reduced effect could be attributed to the unstable DNA conformation around the DNA gaps. Therefore, it is more important to generate controllable overlaps than to produce longer uncontrollable ones.

To investigate the effect of T4 DNA polymerase treatment on DNA assembly, a pUC19 plasmid digested with *Xba*I restriction endonuclease was treated with T4 DNA polymerase, followed by incubation at 37°C for an hour, and subsequently, the resulting DNA mixture was transformed into *E. coli*. Theoretically, the two ss DNA tails generated from T4 DNA polymerase treatment produced no complementary region; they could not anneal to form a circular plasmid. However, colonies contained the re-assembled plasmid pUC19 were observed, clearly demonstrating the occurrences of unspecific annealing in the ss DNA tails (data not shown).

As GC ratios in DNA fragments influence DNA melting temperature and affect the denaturation and annealing processes, all DNA overhangs used in this study were designed with GC ratios ranging from 35% to 65%. It was recommended that the study should design overhangs without extremely high GC ratios, and/or adjust the lengths of overlaps to allow a melting temperature lower than 90°C in case very high GC ratios can not be avoided.

In summary, a nicking endonuclease (NEase) based ligation independent cloning method (NE-LIC) was successfully developed. This method allows the simultaneous assembly of multiple DNA fragments with more freedoms than that of the traditional methods based on type II endonucleases. The NE-LIC could generate controllable ss DNA overlaps compared with any DNA exonucleases that could not do so, it also costs less than USER-LIC does.

## Materials and Methods

### Strains and DNA

*E. coli* strain EC100D *pir*-116 containing plasmid pAH83CI was used for all the DNA assemblies. Plasmid pUKG was used as a template for *attL*-*R6K* and *attR*-*kan* fragments. Chloromycetin gene was cloned from pKD3 plasmid. Plasmid pBHR68 containing *Ralstonia eutropha* PHB synthesis operon was used as a template for cloning *Re* promoter, *phbC*, *phbA*, and *phbB* which encodes *R. eutropha* native promoter, PHA synthase, beta-ketothiolase, and acetoacetyl-CoA reductase, respectively. All strains and plasmids used in this study were listed in Table 1. Detailed primer information for fragments was listed in Table 2.

### Materials and Equipment

Nicking enzymes (Nb.BbvCI, Nt.BspQI and Nb.BtsI), T4 DNA polymerase and T4 DNA ligase used in this study were purchased from New England Biolabs (Ipswich, MA, USA). DNA fragments were amplified using Fast-*pfu* DNA polymerase (TRANSGEN, Beijing, China) and purified by the OMEGA E.Z.N.A. Gel Extraction kit (Omega Bio-Tek, USA). The PCR manipulation of DNA fragments were performed as stated below: after an initial 8 minutes denaturation at 98°C, 5 cycles of 30 seconds at 98°C, 30 seconds at 52°C for annealing, and at 72°C for 15–30 seconds for extension (depending on the length of the fragment), followed by another 30 cycles of PCR amplification with higher annealing temperature as 65°C. Finally an extension step at 72°C for 5 minutes allowed completion of this DNA manipulation process.

### NE-LIC Coupled with *in vivo* Circularization

After the amplification and purification processes, DNA fragments with designed overlaps were digested by 10 U of Nb.BbvCI or Nt.BspQI at 37°C for 1 h. Then the digested fragments were heated to 90°C for 5 min for denaturation, and then chilled to 4°C. The DNA concentrations were determined by Nano Drop Spectrophotometer ND2000 (Thermo Scientific,Wilmington,USA). For NE-LIC coupled with *in vivo* circularization, an equal molar ratio of the fragments was mixed to anneal at 37°C for 1 h into a linear form and then electroporated into competent cells of *E. coli* EC100D *pir*-116 harboring pAH83CI, to allow *in vivo* circularization. Following electroporation, cells were suspended in LB broth and incubated under the following conditions: 30°C for 30 min; 37°C for 30 min and finally another 30°C for 30 min. The cells were then spread onto 10 µg/mL kanamycin petri disk and incubated at 37°C for 16 h. The schematic diagrams are shown in Figures 1A and 1B. The constructs were verified by PCR and DNA sequencing. The buffer for this study was optimized among buffers of T4 DNA ligase, T4 DNA polymerase and Fast-*pfu* polymerase.

### Optimization of Denaturation Temperature

A fragment with two Nb.BtsI sites of 22 bp separated length in the middle was amplified from plasmid pUC19 using primers BtsIf/BtsIr (Table 2), and further purified using DNA OMEGA E.Z.N.A. Gel Extraction kit (Omega Bio-Tek, USA). It was then digested by Nb.BtsI nicking enzyme, and denatured at 70°C, 75°C, 80°C, 85°C, 90°C or 95°C to test the optimal temperature for splitting into two fragments.

### Enhanced Denaturation Efficiency Using Competing Primers

Competing primers that are complementary to the ss DNA released during the denaturation process was added to the denaturation system. Competing primers were designed with the same length of the ss DNA overlaps. The denatured products were purified by OMEGA E.Z.N.A. Gel Extraction kit (Omega Bio-Tek, USA).

### Optimization of the Overlapping Lengths

The 2-fragment NE-LIC coupled with *in vivo* circularization of the R6kγ and Kan fragments was used for the optimization of overlapping lengths. The junction of the R6kγ and Kan fragments was designed to generate different overlaps of 5 bp, 10 bp, 15 bp, 20 bp or 25 bp. Annealing with or without T4 DNA ligase was studied. Electrophoresis of annealing products was performed (Fig. 2).

### Comparisons of Annealing with Gaps and Seamless Annealing

The anneals of R6kγ and Kan fragments with 5 bp, 10 bp, 15 bp, 20 bp and 25 bp complementary overlaps were designed, respectively. For the group with uncontrollable gaps, all the assembled fragments were treated with T4 DNA polymerase at 37°C for 5 min, and then the reaction was terminated using 0.1 vol of 10 mM 2′-deoxycytidine 5′-triphosphate (dCTP). For the group with 2 bp designed gaps, the 2-fragment NE-LIC coupled with *in vivo* circularization of the R6kγ and Kan fragments was used to study the effect. The 2 bp gaps were designed inside the complementary region close to the inner end. For the group without gap, the junction of the R6kγ and Kan fragments was designed to generate overlaps that anneal seamlessly. Electrophoresis of annealing products was performed (Fig. 3).

### PHB Production and Analysis

*E. coli* EC100D *pir*-116 harboring the PHB synthesis operon *phaCAB* constructed by NE-LIC coupled with *in vivo* circularization was incubated at 37°C in LB medium containing (g/L) 5 yeast extract, 10 tryptone and 10 NaCl for 12 h at 200 rpm on a rotary shaker (Series 25D, NBS, New Brunswick, USA). Then they were inoculated into the shake flasks placed on the rotary shaker at 200 rpm placed with 500 ml conical flasks containing 50 ml Terrific Broth (TB) medium containing (g/L) 12 tryptone, 24 yeast extract, 9.4 K_2_HPO_4_, 2.2 KH_2_PO_4_ and 4 ml/L glycerol supplemented with 20 g/L glucose for 48 h [30]. Additionally, 50 mg/L kanamycin was used for maintaining the stability of the plasmids. PHB analysis method was performed as described by Zhou et al [30].
